# Rhythm Generation through Period Concatenation in Rat Somatosensory Cortex

**DOI:** 10.1371/journal.pcbi.1000169

**Published:** 2008-09-05

**Authors:** Mark A. Kramer, Anita K. Roopun, Lucy M. Carracedo, Roger D. Traub, Miles A. Whittington, Nancy J. Kopell

**Affiliations:** 1 Department of Mathematics and Statistics, Boston University, Boston, Massachusetts, United States of America; 2 Center for BioDynamics, Boston University, Boston, Massachusetts, United States of America; 3 Institute of Neuroscience, Newcastle University, Newcastle, United Kingdom; 4 Department of Physiology and Pharmacology, SUNY Health Sciences Center, Brooklyn, New York, United States of America; University College London, United Kingdom

## Abstract

Rhythmic voltage oscillations resulting from the summed activity of neuronal populations occur in many nervous systems. Contemporary observations suggest that coexistent oscillations interact and, in time, may switch in dominance. We recently reported an example of these interactions recorded from in vitro preparations of rat somatosensory cortex. We found that following an initial interval of coexistent gamma (∼25 ms period) and beta2 (∼40 ms period) rhythms in the superficial and deep cortical layers, respectively, a transition to a synchronous beta1 (∼65 ms period) rhythm in all cortical layers occurred. We proposed that the switch to beta1 activity resulted from the novel mechanism of period concatenation of the faster rhythms: gamma period (25 ms)+beta2 period (40 ms) = beta1 period (65 ms). In this article, we investigate in greater detail the fundamental mechanisms of the beta1 rhythm. To do so we describe additional in vitro experiments that constrain a biologically realistic, yet simplified, computational model of the activity. We use the model to suggest that the dynamic building blocks (or motifs) of the gamma and beta2 rhythms combine to produce a beta1 oscillation that exhibits cross-frequency interactions. Through the combined approach of in vitro experiments and mathematical modeling we isolate the specific components that promote or destroy each rhythm. We propose that mechanisms vital to establishing the beta1 oscillation include strengthened connections between a population of deep layer intrinsically bursting cells and a transition from antidromic to orthodromic spike generation in these cells. We conclude that neural activity in the superficial and deep cortical layers may temporally combine to generate a slower oscillation.

## Author Summary

Since the late 19th century, rhythmic electrical activity has been observed in the mammalian brain. Although subject to intense scrutiny, only a handful of these rhythms are understood in terms of the biophysical elements that produce the oscillations. Even less understood are the mechanisms that underlie interactions between rhythms; how do rhythms of different frequencies coexist and affect one another in the dynamic environment of the brain? In this article, we consider a recent proposal for a novel mechanism of cortical rhythm generation: period concatenation, in which the periods of faster rhythms sum to produce a slower oscillation. To model this phenomenon, we implement simple—yet biophysical—computational models of the individual neurons that produce the brain's voltage activity. We utilize established models for the faster rhythms, and unite these in a particular way to generate a slower oscillation. Through the combined approach of experimental recordings (from thin sections of rat cortex) and mathematical modeling, we identify the cell types, synaptic connections, and ionic currents involved in rhythm generation through period concatenation. In this way the brain may generate new activity through the combination of preexisting elements.

## Introduction

The synchronous activity of neural populations results in voltage fluctuations observable in macroscopic (e.g., scalp electroencephalography) and mesoscopic (e.g., local field potential or LFP) recordings. Rhythmic voltage fluctuations—or oscillations—have been observed in the mammalian brain for over a century [1]. Although the purpose of these oscillations remains unknown, neural rhythms appear to temporally organize network activity patterns, and pathological changes in these rhythms often accompany disease [2],[3].

What mechanisms produce these neural rhythms? The complexity of the vertebrate brain—resulting not only from the sheer number of neurons (approximately 10^9^) and their connections (approximately 10^11^ [4]), but also from the many different neuron classes (e.g., the diversity of inhibitory interneurons [5])—affords no simple answers. Yet, simple characteristic structural patterns appear fundamental to the brain's organization [5],[6]. From these elementary network building blocks (i.e., structural and functional *motifs*) more complicated structures may be generated in an efficient way [7]–[9].

Perhaps a similar strategy may be used to understand the rhythmic electrical activity of the brain. For example, the simplest (yet biophysical) model of the 40 Hz (gamma) rhythm involves only two interconnected cells (one excitatory and the other inhibitory) with reciprocal synaptic connections. In this “gamma –motif” the decay of inhibition paces the rhythm [10]–[12]. This motif, and its variations, may be used to construct more complicated gamma networks [13]–[15].

Here we consider how the elementary dynamic building blocks (or dynamic motifs [16]) of the gamma (∼40 Hz) and beta2 (∼25 Hz) rhythms may combine to generate a slower beta1 oscillation. To do so, we develop computational models motivated by a novel method of rhythm generation—period concatenation—as we now describe. We recently observed that application of 400 nM kainate to rat somatosensory cortex in vitro resulted, initially, in distinct rhythms in different cortical layers [17]. In the superficial cortical layers (layers II and III, or LII and LIII) we observed gamma frequency rhythms (38±3 Hz, *n* = 25 observations), and in the deep cortical layers (layer V or LV) nonsynaptic beta2 rhythms (24±2 Hz, *n* = 25 observations). The dominant mechanisms that regulate these two rhythms in secondary cortex are known; the decay time of GABA_A_ IPSPs dictates the gamma period [12], and an outward potassium current in LV pyramidal axons sets the beta2 period [18].

**Figure 1:**
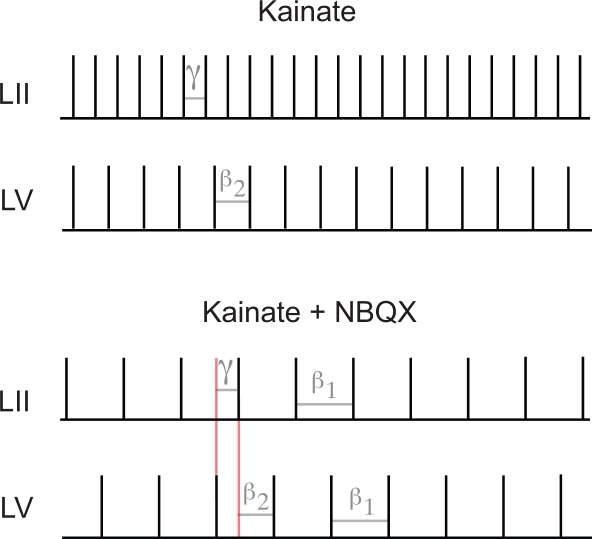
Cartoon illustration of the period concatenation phenomena observed in vitro. In both panels, each vertical line represents a peak in the population activity (or LFP) of the superficial (LII) or deep (LV) cortical layers. Upper panel: under high kainate conditions, oscillations in LII and LV occur at gamma and beta2 frequencies, respectively. Lower panel: following application of NBQX, a beta1 rhythm occurs in both layers. The peaks in oscillatory activity are offset between the layers; a LV peak precedes a LII peak by one gamma cycle (left vertical red line), and a LII peak precedes a LV peak by one beta2 cycle (right vertical red line). The faster rhythms (gamma and beta2) combine to produce the slower beta1 oscillation.

The initial interval of coexistent gamma and beta2 rhythms preceded the transition to a slower beta1 oscillation (15±2 Hz, *n* = 10). This new rhythm appeared in both cortical layers upon reduction of glutamatergic excitation with 2.5 µM NBQX (an AMPA receptor antagonist that also reduces kainate drive [19]). We note that the initial interval of kainate application—and its associated network activity at gamma and beta2 frequencies—was an essential prerequisite to the generation of the beta1 rhythm. If we bathed the slices in kainate and NBQX initially (not kainate alone) then we found no persistent rhythms. Both the population activity (observed in the LFP) and the spiking activity of individual neurons suggested that the beta1 rhythm resulted from period concatenation; the period of the slower beta1 rhythm (∼65 ms) equaled the sum of the periods of the two faster rhythms (gamma ∼25 ms and beta2 ∼40 ms). The ordering of the neural activity in each layer supported the period concatenation hypothesis: in the population and single unit activity of both layers we found delays consistent with individual cycles of the gamma and beta2 rhythms during a single beta1 oscillation. In particular, during the beta1 rhythm, the LII activity followed the LV activity by approximately one gamma cycle (≈1/(40 Hz) or 25 ms), and the LV activity followed the LII activity by approximately one beta2 cycle (≈1/(25 Hz) or 40 ms). To help illustrate these relationships between the cortical layers, we show a cartoon representation of the activity in Figure 1.

The in vitro observations suggest that concatenation of the faster gamma and beta2 rhythms (through a summation of their periods) may generate the slower beta1 oscillation. How might such a concatenation occur? As a simple illustrative model we consider two excitable oscillators, one tuned to spike at gamma frequencies and the other to spike at beta2 frequencies. With enough excitation, both oscillators independently fire at their natural frequencies. If we reduce this excitation and connect the separate oscillators in a particular way, we can generate the slower beta1 rhythm as the concatenation of the gamma and beta2 periods. To do so, we connect the oscillators so that a spike in one resets and causes the other to fire one cycle later. For example, if the beta2 oscillator fires, it resets the gamma oscillator, which then fires 25 ms (one gamma cycle) later. Subsequently, the gamma oscillator spike resets the beta2 oscillator, which then fires 40 ms (one beta2 cycle) later. Connected in this way, both oscillators fire at beta1 frequency; the period (65 ms) is the sum of the natural periods (25 ms and 40 ms) of the excitable oscillators.

Of course, biophysical cells are more complicated than simple excitable oscillators. In what follows, we examine the cell types, intrinsic currents, and synaptic connections that support the beta1 rhythm in vitro. In an ideal model of period concatenation, the mechanisms that support the faster rhythms combine to generate the slower oscillation. We will show that this scheme is nearly—but not quite—met in the computational model. The phenomenon of rhythm generation through period concatenation could be modeled in many different ways (involving, for example, different cell types, ionic currents, and synaptic connections [20].) Here we implement the elementary dynamic building blocks of the gamma and beta2 oscillators (i.e., a gamma motif and beta2 motif) and combine these to generate the beta1 rhythm. In doing so we describe how in vitro observations guide construction of the model and test its predictions. We begin by discussing the coexistent gamma and beta2 activity observed in vitro and simulated in the model. We then describe the transition to beta1 and propose that this transition requires a shift in the deep layer pyramidal cells from antidromic to orthodromic activity. Analysis of the cross-frequency interactions during the beta1 oscillation (without access to the underlying mechanisms) might simply suggest that the slower rhythm modulates the faster activity. As we will show, the combined approach of in vitro recordings, data analysis, and mathematical modeling reveals more detailed information about the intrinsic currents and synaptic connections that generate the beta1 rhythm through period concatenation of the faster oscillations.

## Results

**Figure 2:**
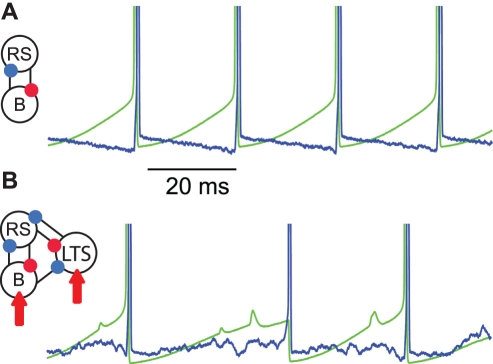
The gamma-motif and its disruption. (A) Reciprocal synaptic connections ( excitatory in red, inhibitory in blue) between a basket cell (B) and RS cell create a single Pyramidal-Interneuron-Network-Gamma (or PING) oscillator. A spike in the RS cell (green trace, spikes truncated) induces a spike in the basket cell (blue trace, spikes truncated). The basket cell then inhibits the RS cell which recovers and fires after approximately 25 ms. The decay time of the inhibitory synapse determines the period of the gamma rhythm. (B) Inclusion of a superficial layer LTS interneuron, and deep layer excitatory inputs (red arrows) to the superficial inhibitory cells, disturbs the gamma-motif.

**Figure 3:**
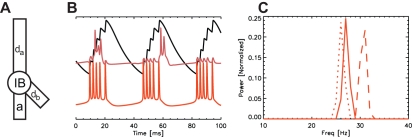
The beta2 reduced model and control of the beta2 rhythm by the M-current. (A) The model of a single IB cell consists of four compartments: an apical dendrite (*d_a_*), a basal dendrite (*d_b_*), a soma (IB), and an axon (*a*). (B) Traces of the voltage in the IB cell axon (orange) and soma (red), and the M-current in the axon (black). Bursts of action potentials in the axon produce spikes and spikelets in the soma, and increase the axonal M-current. Between bursts of action potentials, the M-current slowly decays. The vertical scale in this figure is arbitrary. (C) The normalized power spectra of the voltage in the axon compartment with different scaling of the M-current backward rate function; normal backward rate function (solid curve), decreased backward rate function (dotted curve), and increased backward rate function (dashed curve). The dynamics of the M-current control the interburst interval.

In this section we describe a reduced model of rat somatosensory cortex and the observations that constrain and verify this model. The model consists of four cell types observed in vitro [17]: a regular spiking (RS) pyramidal cell, a basket cell, a low-threshold spiking (LTS) interneuron [21]–[23], and an intrinsically bursting (IB) cell. We show a cartoon representation of the three superficial layer cells and the four-compartment deep layer IB cell in Figures 2 and 3A, respectively. Model chemical and electrical synapses connect these neurons consistent with cortical physiology and observations from the in vitro preparation, as described below. We focus on the intrinsic currents that determine the time scales of each dynamic motif, and on the synaptic connections between cortical layers that disturb and promote each rhythm. In two cases the model predictions are tested in vitro, and in all cases we use the model to suggest more detailed information not observable in experiment. We begin with simulations of the high kainate conditions.

### Superficial Layer Model in High Kainate Conditions

During the high kainate condition in vitro, coexistent gamma and beta2 rhythms occur in the superficial and deep cortical layers, respectively [17]. To model the superficial layer activity, we implement a population of Pyramidal-Interneuron-Network-Gamma (or PING) type oscillators, each consisting of a basket and RS cell. We show the voltage dynamics of a single gamma oscillator (i.e., the gamma-motif) in Figure 2A. The gamma activity results from the interactions between these two cell types, and the decay time of the inhibitory basket cell synapse determines the period of the gamma rhythm [12]. We also include a population of LTS interneurons in the superficial layer [21]–[23]. Under the high kainate conditions, these interneurons fire infrequently and have a disorganizing influence on the gamma oscillator, as we show in Figure 2B. But, following application of NBQX, the LTS interneurons play a vital role in establishing the beta1 motif, as we discuss below. We replicate the RS-basket-LTS circuit (shown in Figure 2B) twenty times and connect the RS neurons with electrical synapses to create a population of superficial layer cells, as described in the Methods section.

### Deep Layer Model in High Kainate Conditions: M-Current Determines Frequency

To model the deep layer activity, we simulate a population of IB cells, each consisting of four compartments: an apical dendrite, basal dendrite, soma, and axon. We show a cartoon representation of a single IB cell model in Figure 3A. Recent experimental and modeling work has shown that the axonal M-current controls the period of the antidromic beta2 oscillation [18]. The same is true in the reduced model presented here; during beta2 activity, bursts of action potentials occur in an IB cell axon and travel antidromically to produce bursts of full action potential spikes and subthreshold “spikelets” in the soma (Figure 3B). The dynamics of the M-current determine the interburst interval [18]. When the axon generates an action potential, the M-current increases. After enough sequential action potentials, the M-current becomes large enough that the outward, hyperpolarizing current prevents further spiking. The M-current then slowly decays and, after sufficient time, another burst of action potentials can begin (Figure 3B). We may increase (or decrease) the interburst interval by decreasing (or increasing) the backward rate function of the M-current (Figure 3C). A decrease in the backward rate function causes the M-current to decay more slowly and the interburst frequency decreases (dotted curve). Conversely, an increase in the backward rate function increases the interburst frequency (dashed curve).

### Deep Layer Model: Anatomy and Connections of the Dendrites

We model the dendrites of an individual deep layer IB cell with two compartments: a basal dendrite and an apical dendrite (Figure 3A). This is, of course, a crude approximation to the true dendritic form. We utilize the two compartments to mimic changes in ionic currents and synaptic inputs that occur across the dendritic structure. In particular, we increase the conductance of hyperpolarization activated currents (h-currents) in the apical dendrite [24],[25], and will connect excitatory NMDA synapses from IB cell axons to IB cell basal dendrites [26],[27] to generate the beta1 rhythm, as we describe below. Two types of inhibitory input also target the IB cell dendrites; the superficial layer LTS interneurons target the ascending apical dendrites, and a Poisson source of IPSPs target the basal dendrites (we provide an interpretation of these random inhibitory inputs below.) Although a crude approximation to the actual pyramidal morphology, we find that this simple model captures the essence of the observed dynamics, as we now describe.

### The Simulated Cortical Column Produces Coexistent Gamma and Beta2 Rhythms

**Figure 4:**
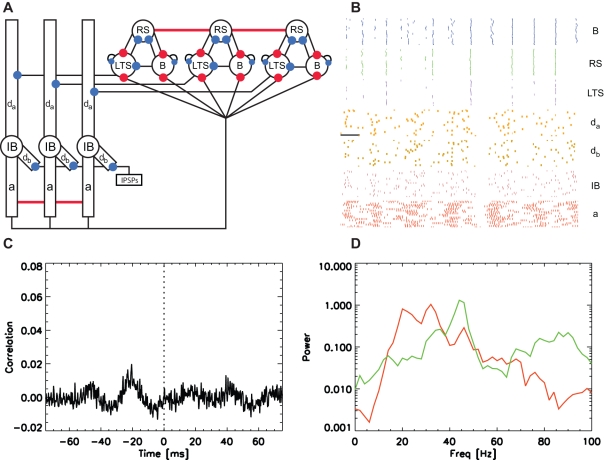
Population model of the high kainate activity. (A) A cartoon representation of the model. The populations in both layers contain twenty replications of each cell, although we only draw three of each cell type in the figure. The deep layer consists of IB cells each with four compartments: *d_a_*, the apical dendrite; *d_b_* the basal dendrite; *IB*, the soma; and *a*, the axon. A Poisson source of IPSPs inhibits each basal dendrite, and we indicate this source with the label IPSPs. The superficial layer contains three cell types: RS cells (RS), basket cells (B), and LTS interneurons (LTS). We denote the termination points of excitatory and inhibitory synapses with red and blue filled circles, respectively. We connect all of the IB cell axons and RS cells with gap junctions (indicated by the horizontal red lines). (B) The spiking activity of the superficial layer cells and deep layer IB cell compartments: B (blue) basket cells; RS cells (green); LTS interneurons (purple); *d_a_* (orange) apical dendrites; *d_b_* (gold) basal dendrites; IB (maroon) somata; *a* (red) axons. Each colored dot represents a spike in a single compartment or cell. The horizontal black line indicates 20 ms. (C) The average cross-correlation between the RS cells and IB cell axons. No obvious correlation structure is apparent. (D) The average power spectra of the RS cells (green) and IB cell axons (red). Coexistent gamma and beta2 activity occur in the superficial and deep layers, respectively.

We show a cartoon representation of the entire reduced model in Figure 4A. We note that ascending excitatory synapses (from the deep layer IB cells to the superficial inhibitory cells) and descending inhibitory synapses (from the LTS interneurons to the IB cell apical dendrites) connect the two layers. In Figure 4B we show a typical simulation result for the 80 cells in the model (twenty of each type). We plot the spiking activity of the three superficial layer cell types, and the spiking activity of the dendrites, somata, and axons of the IB cell population. We find that the individual IB cell dendrites generate action potentials infrequently and remain in a mostly inactive state. The IB cell axons generate bursts of action potentials at beta2 frequencies that are weakly synchronized across the population. The weakly organized beta2 activity results in a noisy synaptic input to the superficial layer inhibitory cells. The result is a disorganization of the dynamic motifs that define the gamma rhythm; the basket cells are perturbed directly by the deep layer excitatory inputs, while the the RS cells are perturbed indirectly through the deep layer excitation of the LTS interneurons. We illustrated the effects of these disorganizing deep layer inputs on the gamma rhythm in Figure 2B and will show below that removing the noisy synaptic inputs from the IB cells increases the superficial layer gamma power.

We plot in Figure 4D the population average power spectra (see Methods section) of the RS cells (green curve) and IB cell axons (red curve) and find broad spectral peaks in the gamma range (40–50 Hz) in the superficial layer and in the beta2 range (20–30 Hz) in the deep layer. We also show the average cross-correlation (see Methods section) between the spike times of the IB cell axons and RS cells in Figure 4C. We find no obvious correlation between the activity of the two layers. Thus, although the layers interact through chemical synapses, these interactions are too weak to correlate the spike times of the two layers. In particular, the disorganizing beta2 frequency input from the IB cells to the superficial layer inhibitory cells does not phase lock (and therefore correlate) the rhythmic activity of the two layers. That the model generates coexistent gamma and beta2 rhythms is not surprising—we include in the model components necessary to produce these two rhythms and allow only weak interactions between the two layers.

Having established that the reduced model can generate gamma and beta2 oscillations, we now determine how these model rhythms respond to a series of experimental manipulations performed in vitro. In each challenge the experimental results test and constrain the computational model. In addition, we use the model to suggest more detailed information not accessible in experiment. Our manipulations focus on the synaptic connections and intrinsic currents that support (or disturb) each rhythm.

### Separating Cortical Layers Boosts Superficial Gamma Power

**Figure 5:**
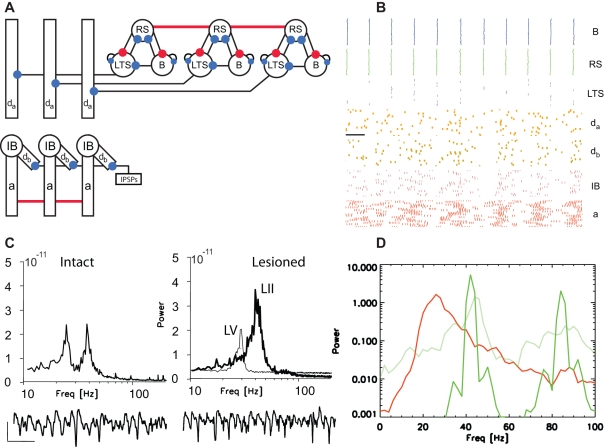
Population model of the high kainate activity with cortical layers separated. (A) Cartoon representation of the model. The ascending synapses and the apical dendritic compartments of the IB cells have been removed. (B) The spiking activity of the superficial and deep layer cells in the model. We note the increased synchrony of the superficial layer activity compared to that observed with layers intact. (C) Power spectra of the intact and lesioned in vitro slice preparation. In the former, LFP data were recorded from intermediate cortical layers. In the latter, we show the power spectra of LFP data recorded from deep layers (LV) and superficial layers (LII). The power is expressed in units of V^2^/Hz. Below each power spectrum we show example LFP traces from intermediate (left) and superficial (right) cortical layers. The vertical and horizontal lines at the bottom left indicate 50 µV and 100 ms, respectively. (D) The average power spectra of the RS cells (dark green) and IB cell axons (red) in the model following the separation of layers. For comparison, we also plot the average RS power spectrum for the intact model (light green). The superficial layer gamma power increases following the separation of the layers.

Analysis of in vitro slice preparations revealed a statistically significant increase in the gamma power of the superficial layers (215±29%, *n* = 6, *P*<0.05) following a lesion through layer IV. We show example LFP recordings and power spectra for these in vitro results in Figure 5C. These observations suggested some functional connectivity between LV cells and superficial layer neurons involved in generating the gamma oscillation [17].

In the computational model, the superficial layer PING oscillators produce the gamma rhythm. We expect that ascending excitatory synapses from the IB cell axons perturb these superficial layer oscillators (both directly and through activation of superficial LTS interneurons) and therefore disturb the gamma rhythm. To test this expectation we separate the cortical layers in the model and observe the resulting activity. Specifically we remove the ascending excitatory synapses from the IB cells to the inhibitory cells, and we disconnect the apical dendrites from the IB cells (we assume that a slice through layer IV severs the ascending dendrites of the IB cells.) We suggest the associated parameter changes in Figure 5A. Following separation and elimination of the disorganizing inputs, the spiking activity of the superficial PING oscillators becomes more synchronized (Figure 5B). This increased synchronization boosts the superficial gamma power (Figure 5D), in agreement with the in vitro results.

In experiment, separating the superficial and deep cortical layers requires a resection through the intervening layer IV. This physical separation necessarily destroys all interlaminar connections. In the model, we may study specific interlaminar connections to determine those that most disturb the superficial gamma rhythm. To do so we remove, one by one, the three types of interlaminar connections: (i) excitatory synapses from the IB axons to the basket cells, (ii) excitatory synapses from the IB axons to the LTS interneurons, and (iii) the apical dendritic compartments of the IB cells. We find that eliminating the first two connections (but not the last) increases the superficial gamma power (data not illustrated). The excitatory input from the IB cells disturbs the gamma oscillators directly by depolarizing the basket cells, and indirectly by exciting the LTS interneurons (which spike and hyperpolarize the RS cells.) Without the disorganizing effects of the deep layer input, the RS cells drive the population of PING oscillators in synchrony.

### Blocking IPSPs Boosts Deep Beta2 Power

**Figure 6:**
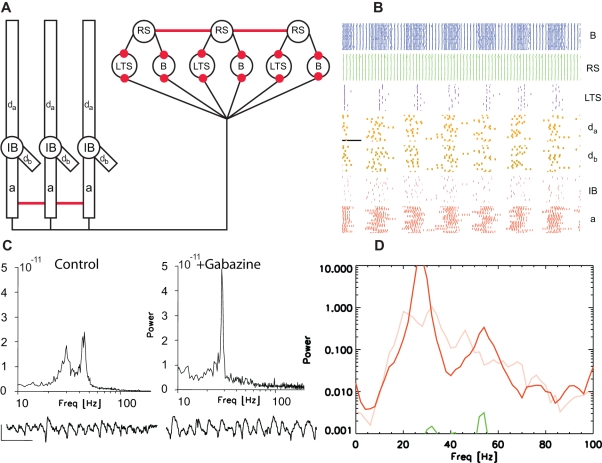
Population model of the high kainate activity with IPSPs blocked. (A) Cartoon representation of the model with all inhibitory synapses removed. (B) The spiking activity of the superficial and deep layer cells in the model. We note the increased synchrony of the deep layer activity compared to that observed under normal conditions. (C) Power spectra of the in vitro slice preparations under control conditions and following application of 250 nM of gabazine. The power is expressed in units of V^2^/Hz. Below each power spectrum we show example LFP traces; the vertical and horizontal lines at the bottom left indicate 50 µV and 100 ms, respectively. (D) The average power spectra of the RS cells (green) and IB cell axons (dark red) in the model following IPSP block. For comparison, we also plot the average IB power spectrum in the original model (light red). The deep layer beta2 power increases following the elimination of all IPSPs.

Inhibition plays a vital role in pacing the gamma and beta1 rhythms [12],[17]. To determine the effect of inhibition on the beta2 oscillation, we applied 250 nM of gabazine to the in vitro slice preparation and found a statistically significant (*n* = 5 observations, *p*<0.05) increase in the deep layer beta2 power (Figure 6C) consistent with previous results [18].

In the model, IPSPs (from the superficial layer LTS interneurons and deep layer random sources) target the IB cell dendrites. We think of the random inhibitory inputs as representing a noisy motif, perhaps important for other deep layer rhythms, but disruptive to the beta2 activity. Therefore, we expect that blocking IPSPs will eliminate these inputs and reduce the orthodromic, disruptive influence on the antidromic, beta2 rhythm. We determine the effect of blocking IPSPs in the model by setting the conductance of all inhibitory synapses to zero, as we indicate in Figure 6A. We find that elimination of IPSPs in the model reduces the orthodromic disruption of the beta2 rhythm. The IB cell dendrites now burst in response to the antidromic propagation of axonal activity. Without the disruptive inhibitory input to the IB cell dendrites, the IB cell axons burst with greater synchrony (Figure 6B), and thus boost the beta2 power (Figure 6D) in agreement with the experimental results. We note that removing the basket cell IPSPs causes the electrically coupled RS cells to establish a positive feedback loop and fire rapidly (Figure 6B). In the simple model of the gamma -motif implemented here, the RS cells spike on each cycle of the gamma rhythm. In vitro, individual RS cells spike much more sparsely during gamma activity [17]. We do not model this sparse activity here, which would require a much larger population of RS cells [12],[13]. Therefore the model of superficial layer activity is not valid after removing the IPSPs that pace the gamma rhythm.

We also use the model to investigate the type of inhibitory synapse most disruptive to the beta2 rhythm. To do so, we determine the individual effects of removing inhibitory synapses from: (i) the basket cells alone, (ii) the LTS cells alone, and (iii) the random sources (to the IB dendrites) alone. We find that, of the three, removing the latter two increases the beta2 power (simulations not illustrated). Again we conclude that eliminating the disruptive activity of the IB cell dendrites (here by removing disruptive inhibitory inputs to these compartments) boosts the beta2 activity.

### Blocking h-Current Boosts Deep Beta2 Power

**Figure 7:**
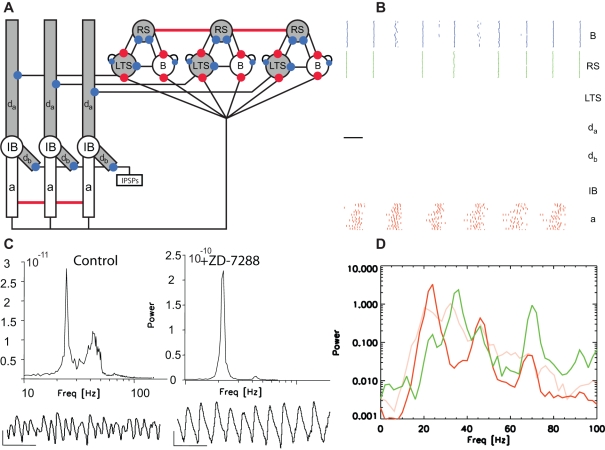
Population model of the high kainate activity following h-current block. (A) Cartoon representation of the model with h-current removed from the cells and compartments shaded gray. (B) The spiking activity of the superficial and deep layer cells in the model. We note the increased synchrony of the deep layer activity compared to that observed under normal conditions. (C) Power spectra of the in vitro slice preparations under control conditions and following application of 10 µM ZD-7288. The power is expressed in units of V^2^/Hz, and we note the change of scale in the two figures. Below each power spectrum we show example LFP traces; the vertical and horizontal lines at the bottom left (right) indicate 50 µV (100 µV) and 100 ms, respectively. (D) The average power spectra of the RS cells (green) and IB cell axons (dark red) in the model following h-current block, and without h-current block (light red). The deep layer beta2 power increases following h-current blockade.

The beta2 rhythm in the deep layer IB cell population originates in the axonal compartments (in fact, the axonal M-current sets the period of this rhythm [18]). Input from the IB cell dendrites disturbs this rhythm, and we expect that silencing the dendritic compartments would boost the deep layer beta2 power. We test this hypothesis in the mathematical model by blocking a current important to both the dendrites and their superficial layer inputs (the LTS interneurons): the h-current. We do so by setting the h-current conductance to zero in the RS cells, LTS interneurons, and IB cell dendrites. We shade the affected cells and compartments gray in Figure 7A and plot an example of the model spiking activity in Figure 7B. We find that blocking all h-currents eliminates the disruptive effect of the IB cell dendrites on the beta2 rhythm. The IB cell axons burst with increased synchrony and therefore the beta2 oscillations in the IB cell population increase in magnitude. The result is an increase in beta2 power, as we show in Figure 7D. We tested the effect of h-current block in vitro by applying 10 µM ZD-7288 to the slice preparation and found a dramatic increase in the beta2 power (Figure 7C). This increase was statistically significant (*n* = 5 observation, *p*<0.05) and verified the model prediction.

The global blockade of h-current does not suggest which particular cell type (if any) is most important to this effect. Therefore, we consider in the model the effects of h-current block: (i) in the RS cells alone, (ii) in the LTS interneurons alone, (iii) in the IB cell basal dendrites alone, and iv) in the IB cell apical dendrites alone. Of these three, only the middle two increase the beta2 power. By eliminating the h-current in the IB cell basal dendrites, we essentially silence these compartments and reduce the effects of the random inhibitory synaptic inputs to them. By eliminating the h-current in the LTS interneurons, we silence these cells and their disturbing inputs to the IB cells. We conclude that eliminating the disruptive inputs to the IB cells enhances the deep layer (antidromic) beta2 rhythm.

### Strengthening NMDA Synapses between IB Cells Preserves the Fast Rhythms

In vitro, the initial interval of coexistent gamma and beta2 rhythms must precede the slower beta1 activity. Without this initial interval (i.e., with immediate application of NBQX to the slice preparation) no beta1 activity occurs. We therefore expect that the coexistent fast rhythms change the network in a way that supports the slower beta1 oscillation. In addition, we know that NMDA receptor-mediated synaptic events support the beta1 activity—blocking NMDA before or after NBQX application prevents the beta1 oscillations (as we discuss in detail below). To model the change in network structure that occurs during the fast rhythms, we assume a strengthening of all-to-all NMDA synapses from each IB cell axon to all IB cell basal dendrites [26],[27]. We note that potentiation of NMDA synapses has been observed in LV pyramidal cells with bursting behavior [28].

We expect that these NMDA synapses between the IB cells strengthen gradually during the interval of coexistent gamma and beta2 activity. What effect does this gradual change have on the faster rhythms? In vitro we observed that the patterns of field potentials remained constant from the onset of gamma and beta2 activity to immediately before application of NBQX. Whatever changes occurred in the network to support the beta1 activity had no impact on the faster rhythms. We test this in the model by strengthening the NMDA synapses between the IB cell population under the high kainate conditions. In agreement with the experimental observations, we find no change in the gamma and beta2 activity of each layer; the power spectra in the deep and superficial layers match those shown in Figure 4D (results not shown.) We conclude that the strengthening NMDA synapses between the IB cells do not impact the network dynamics until a reduction of excitation (induced by NBQX) occurs in the model, as we now describe.

### Low Kainate Drive: Synchronous Beta1 Rhythm

After an initial interval of coexistent gamma and beta2 rhythms in vitro, the transition to beta1 activity followed application of NBQX, resulting in a reduction of glutamatergic excitation via AMPA and kainate receptor subtypes. In both the deep and superficial layers, population activity (as observed in the LFP) oscillated at beta1 frequency and a consistent lead/lag relationship appeared between the two layers: LII activity preceded LV activity by ∼40 ms, and LV activity preceded LII activity by ∼25 ms (see Figure 1). We note that the timing of these lead / lag relationships suggests that the faster dynamics motifs (i.e., the gamma-motif [25 ms period] and beta2-motif [40 ms period]) collaborate to generate the beta1 rhythm [17].

**Figure 8:**
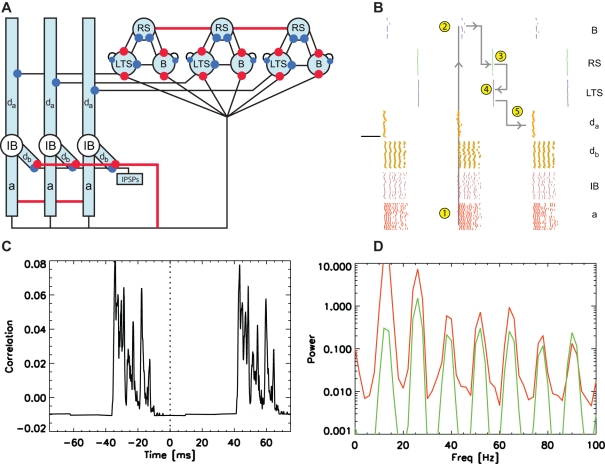
Population model of the low kainate drive activity following an interval of coexistent gamma and beta2 rhythms. (A) To represent the reduced excitation in the model, we hyperpolarize the cells and compartments shaded blue. We include NMDA synapses extending from each IB cell axon to all IB cell basal dendrites; we show these synapses in red. (B) The spiking activity of the three superficial layer cells, and of the dendrites, somata, and axons of the IB cell population. The numbers within yellow circles and gray lines indicate the steps in the beta1 rhythm as it propagates from the deep to superficial layer and back. (C) The average cross-correlation between the RS cells and IB cell axons. (D) The average power spectra of the RS cells (green) and IB cell axons (red).

We now consider whether such collaborative interlaminar interactions can produce the beta1 oscillation in the model. We first assume that reduction of glutamatergic excitation decreases excitation in the network and inactivates the gamma and beta2 motifs [29]. We approximate this reduction by decreasing the depolarizing input current to the superficial layer cells, and the deep layer IB cell dendrites and axons. We also halt the Poisson distribution of IPSPs to the IB cell dendrites, thereby eliminating these disruptive inputs. We indicate these changes in Figure 8A by shading blue the affected cells and compartments. As described above, we also include NMDA synapses (decay time constant 100 ms) within the deep layer IB cell population that target the IB cell basal dendrites; we indicate these slowly decaying excitatory synapses with red lines and filled circles in Figure 8A.

### Mechanisms of the Beta1 Rhythm: Simulation Results

Producing the beta1 rhythm requires many mechanisms that support the superficial gamma and deep beta2 activity. To describe these mechanisms, we follow a cycle of the simulated oscillation as it propagates from the deep to superficial layer and back (Figure 8B). We start at a burst of activity in the IB cell axons (red dots, see Label 1 in Figure 8B). This burst delivers strong excitatory input to the superficial layer inhibitory cells. The basket cells spike (blue dots, Label 2 in Figure 8B), thus inhibiting the RS cells and the LTS interneurons. We note that the basket cells fire and inhibit the LTS interneurons before the ascending NMDA synapses can depolarize these interneurons. This occurs in the model because we make the rise time of the NMDA synapse longer for the LTS interneurons than for the basket cells (see Methods section).

After receiving inhibitory input, the h-currents of the RS cells activate and depolarize these cells on a slow time scale. The RS cells recover from inhibition and spike (green dots, Label 3 in Figure 8B), inducing the population of LTS interneurons to fire. Only a weak excitatory input from an RS cell is required to push an LTS interneuron past spike threshold; the slowly decaying excitatory input from the deep layer and the intrinsic h-current have steadily depolarized each LTS interneuron. Upon spiking (purple dots, Label 4 in Figure 8B), the LTS interneurons inhibit the RS cells and the IB cell apical dendrites, temporarily halting the slow depolarization of the deep layer cells due to NMDA synaptic input. The slow depolarization of the dendrites continue—due to both h-currents and NMDA synapses—and the IB cells spike (Label 5 in Figure 8B), inducing a synchronous burst in the deep layer population and restarting the beta1 cycle.

Computing the population average power spectra of the deep and superficial layer cells, we find peaks near 13 Hz and higher order harmonics (e.g., 26 Hz, 39 Hz, 52 Hz, and so on; Figure 8D). For the RS cell, we note that the largest peak occurs near 26 Hz. The power of this peak includes two contributions: (1) harmonic power of the 13 Hz oscillation (note 2×13 = 26 Hz), and (2) power resulting from the interval between an RS cell spike and the subsequent inhibitory input (one beta2 cycle).

The model of beta1 activity agrees with the observed rhythm in a fundamental way: this rhythm results from period concatenation. To show this we plot in Figure 8C the population average cross-correlation between the spiking activity of the RS cells and the IB cell axons. We find two intervals of increased correlation: between approximately −35 ms and −20 ms, and between approximately 45 ms and 60 ms. Thus, the deep layer population activity precedes the superficial layer activity by approximately 30 ms. The reason for this delay is that the bursting IB cell axons activate the superficial basket cells that inhibit the superficial RS cells. We also conclude that the superficial pyramidal activity precedes the deep layer activity by approximately 50 ms. The reason for this delay is that the RS cells activate the superficial LTS interneurons which inhibit the deep IB cell apical dendrites. These correlation results show that the temporal distributions of the superficial and deep layer activities in the model agree with those of the experiments. In both the model and experiments, during the beta1 rhythm the LII activity follows the LV activity by approximately one gamma cycle (30 ms or ≈33 Hz), and the LV activity follows the LII activity by approximately one beta2 cycle (50 ms or ≈20 Hz) [17].

### Mechanisms of the Beta1 Rhythm: Role of Intrinsic Currents

We note that the rhythmic activity of the model IB cells differ during the high kainate and low kainate drive conditions. During high kainate conditions, antidromic activity generates the beta2 rhythm. The M-current in the axons sets the period of the IB cell bursting [18], and the dendritic activity interferes with this rhythm. During low kainate drive conditions, following potentiation of the NMDA synapses, orthodromic activity generates the beta1 rhythm. The h-current contributes to the dendritic depolarization following inhibitory input from the superficial layer and induces the axons to fire, thus continuing the beta1 oscillation.

**Figure 9:**
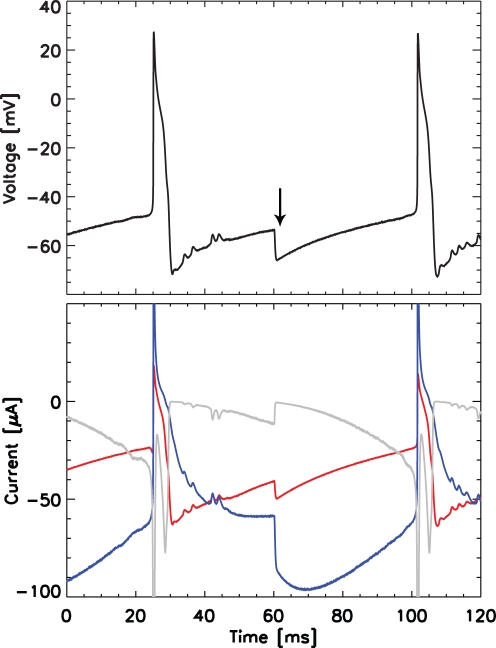
Voltage of the apical dendrite (upper) and intrinsic or synaptic currents (lower) in the basal and apical dendrites of an IB cell during beta1. In the lower figure we show the h-current (blue) and the fast sodium current (gray) of the apical dendrite, and the NMDA synaptic current (red) to the basal dendrite. Negative values indicate inward currents. Inhibitory input from an LTS interneuron arrives at the arrow (upper panel) and activates the h-current of the apical dendrite (lower panel) but has little effect on the NMDA current of the basal dendrite.

The intrinsic currents of, and synaptic inputs to, the IB cell dendrites play an important role in the beta1 activity. We illustrate the dynamics of these currents and inputs within the dendritic compartments of a single IB cell in Figure 9. During a burst of activity in the IB cell axons (e.g., a strip of red dots in Figure 8B) the slowly-decaying NMDA current (red in Figure 9) activates and depolarizes the basal dendrite (near *t* = 25 ms in Figure 9). Approximately 30 ms later, inhibitory synaptic input from a superficial LTS interneuron hyperpolarizes the apical dendrite (arrow, upper figure) and activates the apical dendritic h-current (blue). Both the h-current and NMDA current continue to depolarize the IB cell until a fast sodium current quickly activates (gray) and the neuron fires a burst of spikes near *t* = 100 ms. During spiking, dendritic calcium and potassium currents activate (not shown), the h-current and NMDA current reset, and the beta1 cycle restarts. We note that the slowly decaying NMDA input depolarizes the basal dendrites, and that the population of dendrites enters a more active state than during the high kainate conditions (compare the dendritic activity shown in Figures 4 and 8). The synchronous bursts of activity strengthen the postsynaptic effect of the IB cells and effectively strengthen the ascending synapses from the deep to superficial layer.

**Figure 10:**
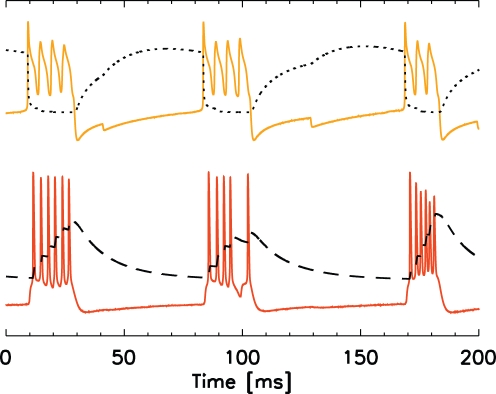
Interplay of the M-current and h-current during beta1. Traces of the voltage in the IB cell apical dendrite (yellow) and axon (orange), the h-current gating variable in the apical dendrite (dotted curve), and the M-current gating variable in the axon (dashed curve). Vertical scale arbitrary.

To illustrate the interplay of the M-current and h-current during beta1, we plot in Figure 10 examples of the apical dendritic voltage (yellow) and axonal voltage (orange) of an IB cell, and the gating variables for the h-current (dotted curve) and M-current (dashed curve). When the apical dendrite receives an IPSP (from a superficial LTS interneuron), the h-current gating variable opens and depolarizes the dendrite, thus promoting spiking. When the IB cell generates an action potential, this gating variable closes and removes this depolarizing influence. The M-current in the axon behaves in an opposite way. When the axon bursts, this gating variable opens and hyperpolarizes the axon, thus preventing further spiking. The axon may spike again only after the M-current decays sufficiently. This example illustrates the complementary effects of the M-current and h-current in the IB cell model. The M-current acts to prevent spiking in the axon, while the h-current acts to promote spiking in the dendrite.

In an ideal model of period concatenation, the mechanisms that generate the two fast rhythms would combine to produce the slow oscillation. This statement is nearly—but not quite—appropriate for the model proposed here. In the model of superficial layer activity, the inhibitory synapse from the basket cell to the RS cell paces the gamma rhythm. Increasing the decay time of this synapse slows the gamma rhythm. A population of these basket cell synapses participates in the beta1 rhythm, and if we increase the decay time of these synapses, then we also slow the beta1 oscillation. Thus, a fundamental mechanism pacing the fast gamma rhythm—namely the basket cell inhibitory synapses—also paces the beta1 rhythm. A more complicated relationship exists between the beta2 rhythm and its contribution to beta1. During beta2 activity, M-currents in the IB cell axons pace the deep layer rhythm. But, during beta1 these M-currents have less influence on the frequency of the deep layer activity. More important are the h-currents, excitatory synaptic inputs, and inhibitory synaptic inputs to the IB cell dendrites. The transition from beta2 to beta1 involves a switch in the IB cell from antidromic to orthodromic activity. Therefore the beta2 component of the beta1 rhythm is dominated by mechanisms in the IB cell dendrites, not the IB cell axon. Thus, in the model, the concatenation depends on having two mechanisms (an M-current and an h-current combined with excitatory and inhibitory synaptic inputs) with the same time scale. In the model, the same cells generate the coexistent fast rhythms and combined slow oscillation, although the biophysical mechanisms important to each rhythm change in the deep layer IB cells. Simpler period concatenation models may be developed, but we do not believe that these models would agree in all ways with the in vitro data.

In what follows, we compare the model with an additional set of experimental manipulations. We show that the two are consistent and verify a model prediction of the fundamental role for the h-current in vitro. We also use the model to suggest specific mechanisms important to the beta1 activity and its propagation between cortical layers. We begin with the observation that:

#### Separating cortical layers destroys beta1 activity

**Figure 11:**
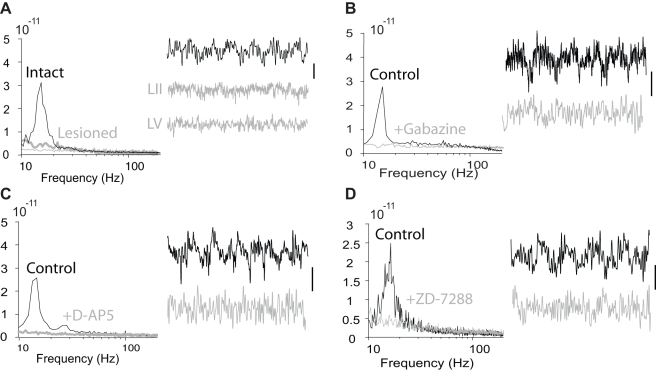
Results from in vitro slice recordings during low kainate drive conditions. In each case, we show the power spectra (in units of V^2^/Hz) computed during control conditions and: (A) after a lesion through layer IV, (B) after ISPSs blocked, (C) after NMDA blocked, and (D) after h-current blocked. To the right of each power spectrum we plot 500 ms of recorded data during the control (black) and manipulated (gray) conditions; the vertical black lines indicate 50 µV. In each case, the experimental manipulation reduces the beta1 power.

In vitro separation of the deep and superficial cortical layers abolished the beta1 rhythm [17]. We show these results here in Figure 11A, where we plot example LFP recordings and power spectra from the slice preparation. A lesion through layer IV in vitro revealed a statistically significant decrease in the beta1 power (*n* = 5 observations, *p*<0.05).

In the model, beta1 activity involves interactions between the deep and superficial layers. We therefore expect that removing interlaminar connections should destroy the rhythm. To test this we mimic a lesion through layer IV in the model by eliminating the ascending excitatory input from the IB cells to the superficial inhibitory cells, and disconnecting the apical dendritic compartments of the IB cells. We find that this separation does indeed destroy the beta1 rhythm in agreement with the in vitro results. After this change we find no activity in either cortical layer of the model. (Here, and in what follows, we do not show the simulation results; the manipulations eliminate or greatly reduce the beta1 activity in the model, and the associated figures—of limited spiking activity or flat power spectra—are not informative.) The beta1 activity stops for one primary reason in the model: the rhythm cannot propagate between the cortical layers without interlaminar connections.

We also use the model to examine the effects of individual interlaminar interactions during the beta1 rhythm. We find that eliminating the ascending synapses from the IB cells to the superficial inhibitory neurons reduces the beta1 activity, and that disconnecting the IB cell apical dendrites destroys the beta1 rhythm. We note that these apical dendrites connect the superficial to deep layer through the inhibitory LTS synapses, which act to depolarize the apical IB dendrites (through activation of an h-current). Without these sources of excitation to the IB cells, the beta1 rhythm cannot continue.

#### Blocking IPSPs destroys beta1 activity

The IPSPs serve a fundamental role in generating the beta1 rhythm [17]. To test the effects of IPSP block on beta1 in vitro we applied 250 nM gabazine to the slice preparation and observed an abolition of the modal peak in the spectrum in the beta1 frequency band (Figure 11B). This decrease was statistically significant (*n* = 5 observations, *p*<0.05).

In the model the beta1 rhythm propagates from the superficial to deep cortical layer through inhibitory synapses (from the LTS interneurons to the IB cell apical dendrites). We therefore predict that blocking IPSPs should eliminate the descending inputs and disrupt the beta1 rhythm. We test this prediction in the model by setting the conductance of all inhibitory synapses to zero, as we indicated in Figure 6A. These parameter changes eliminate the beta1 rhythm in the model (results not shown) in agreement with the in vitro data.

We find that, in the model, removing only the basket cell synapses reduces the beta1 activity, and that removing only the LTS interneuron synapses destroys the beta1 activity. In the former case, loss of the basket cell synapses removes the mechanism pacing the beta1 rhythm in the superficial layer. Without this mechanism, cells in both layers continue to spike, but in a disorganized manner, therefore reducing the beta1 power. In the latter case, the LTS interneurons do not form inhibitory synapses on the IB cell apical dendrites and the dendritic h-currents—necessary to depolarize the IB cell dendrites—remain inactive. The IB cells no longer burst and, without their excitatory postsynaptic effects, the entire network remains inactive.

#### Blocking NMDA destroys beta1 activity

In vitro the transition to beta1 required an initial interval of coexistent gamma and beta2 activity [17]. Following gamma and beta2 rhythm generation, when AMPA and kainate receptor-mediated activity was reduced with NBQX, beta1 activity occurred. NMDA receptors are essential to this activity; blockade of NMDA receptors with D-AP5 (50 µM) abolished the beta1 population rhythm (Figure 11C, *n* = 6, *p*<0.05).

In the model we proposed that strengthening NMDA receptor-mediated synapses between the IB cell population supported the beta1 rhythm. It did so by providing a slow depolarization—and increased excitability—to IB cell basal dendrites. To test the role of this current in the model we remove these excitatory synapses from the IB cell axons to the IB cell basal dendrites. The effect of this parameter change is to hyperpolarize the IB cell basal dendrites and dramatically reduce the spiking activity in the IB cell population. This hyperpolarization and reduced connectivity also increases the interburst interval of the IB cell axons and desynchronizes their output. The desynchronization results in weak synaptic input to the superficial layer. Both the superficial and deep layers continue to spike at a slower rate and in a more disorganized way (simulations not illustrated), eliminating the beta1 power in both layers, in agreement with the in vitro results.

#### Blocking h-current destroys beta1 activity

We proposed above that the h-current plays a vital role in maintaining the beta1 rhythm; the LTS interneurons inhibit the IB cell apical dendrites and activate this depolarizing current to continue the beta1 activity. We therefore predict that, in contrast to the potentiating effect of blocking the h-current on the beta2 rhythm (Figure 7), blocking the h-current should disrupt the beta1 oscillation. To test this in the model we set the h-current conductance to zero in all cells and compartments indicated in Figure 7A. We find that blocking all h-currents in the model eliminates the beta1 rhythm (results not shown). We test the effect of h-current block in vitro by applying 10 µM ZD-7288 to the slice preparation and find a ten-fold decrease in the beta1 power (Figure 11D). This decrease is statistically significant (*n* = 6 observations, *p*<0.05) and validates the model prediction.

We find the same result in the model if we block the h-current in the RS cells alone, in the LTS interneurons alone, in IB cell basal dendrites alone, or in the IB cell apical dendrites alone. We note that the h-current block results in a hyperpolarization—and therefore inactivation—of the affected cells or compartments. We conclude that inactivation of either the IB cell dendrites, RS cells, or LTS interneurons eliminates the beta1 rhythm. The former two populations provide the excitatory drive necessary to continue the beta1 rhythm through their postsynaptic effects. The latter population depolarizes the IB cell apical dendrites through activation of an h-current.

### Descending NMDA Synapses

Analysis of the in vitro data allowed us to constrain the computational model in many ways, but not completely. In the model of beta1 activity described above, we considered a strengthening of NMDA synapses between the population of deep layer IB cells. As a second model for the change in network connectivity that supports the beta1 rhythm, we consider NMDA synapses that *descend* from the superficial to deep layer pyramidal cells. In this case, we include slow excitatory synapses (rise and decay times of 10 ms and 150 ms, respectively) from each superficial layer RS cell to all IB cell apical dendrites [15]. Performing simulations identical to those described above, we find that the model produces beta1 activity with the characteristics of period concatenation. Moreover, we find that separating the cortical layers, blocking IPSPs, blocking NMDA synapses, or blocking the h-current destroys the beta1 activity. Thus, this second model—with potentiating NMDA synapses originating in the superficial layer RS cells and targeting the deep layer apical dendrites—also produces results consistent with the in vitro experiments and modeling results described above.

### A Simpler Model

The essential ideas of the previous model can be captured in a simpler model, but at the expense of becoming more abstract. To construct a simpler model, we do not replicate the cells and dynamic motifs to create neural populations. Instead, we employ single copies of the gamma-motif, the beta2 -motif (a single IB cell), and the LTS interneuron. In addition, we represent the IB cell dendrite as a single compartment (and do not distinguish between the apical and basal dendrites.) In each cell and compartment we implement the same currents and synaptic connections utilized in the more detailed model (e.g., an M-current in the IB cell axon and ascending excitatory synaptic connections from the IB cell to the superficial inhibitory cells.) The goal of creating such a simple model is to understand the fundamental mechanisms that support the beta1 oscillation.

**Figure 12:**
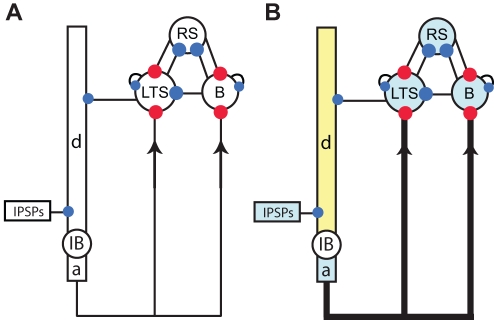
Cartoon representations of the single cell models. (A) The deep layer consists of a single IB cell with three compartments and a Poisson source of IPSPs. The superficial layer consists of three cells: RS, the RS cell; B, the basket cell; LTS, the LTS interneuron. (B) To represent the low kainate drive conditions, we hyperpolarize the cells and compartments colored blue. To represent the strengthening of NMDA synapses, we depolarize the dendritic compartment of the IB cell (yellow) and increase the strength of the ascending synapses (drawn with thickened black lines).

We show a cartoon representation of the simple model in Figure 12A. In the superficial layer, the RS and basket cells form a single (PING) oscillator that generates gamma activity, and in the deep layer a single IB cell axon generates the beta2 rhythm. The mechanisms that support the gamma- and beta2-motifs are identical to those in the more detailed model. We simulate the transition to beta1 activity in two ways. First, we decrease the excitability of the network by reducing the depolarizing input currents to all cells and compartments except the IB cell dendrite and soma; we indicate these hyperpolarizations by shading the affected cells and compartment blue in Figure 12B. Second, we depolarize the IB cell dendrite (yellow) and strengthen the ascending synapses from the IB cell to superficial inhibitory cells (thick black lines, Figure 12B). Both changes mimic effects in the more detailed model. For the population of cells described above we included excitatory NMDA synapses between the IB cells. These synapses act to depolarize the IB cell basal dendrites and help synchronize the IB cell bursting, thus effectively strengthening the ascending synapses. In the simple model, we approximate the effect of increased IB cell synchronization as increased ascending synaptic input. The result is stronger synapses from excitatory to inhibitory cells, which we might also interpret as potentiation of these synapses between the two cell populations. We find that the simple model can reproduce all of the in vitro observations (e.g., blocking the h-current boosts beta2 activity and eliminates beta1 activity; data not shown). We suggest that this greatly reduced model reveals the fundamental mechanisms of the rhythms and period concatenation with four (rather than 80) cells.

## Discussion

We have constructed a computational model, consisting of four cell types, and compared it with in vitro observations from rat somatosensory cortex [17]. In those recordings, we observed a transition from coexistent gamma and beta2 rhythms in the superficial and deep cortical layers, respectively, to a common beta1 oscillation in both cortical layers. We proposed that the slower rhythm resulted from the concatenation of periods of the two faster rhythms: gamma period (25 ms)+beta2 period (40 ms) = beta1 period (65 ms). Both the fast and slow rhythms were sensitive to numerous experimental manipulations (e.g., separating the cortical layers boosted the gamma power under high kainate conditions and eliminated the beta1 power under conditions of low kainate drive.)

In this manuscript, we showed that a biologically realistic, yet simplified, computational model could reproduce the coexistent gamma and beta2 rhythms, the transition to beta1, and numerous experimental manipulations. Moreover, we used the model to suggest the specific mechanisms important for the generation and modification of each rhythm. In the superficial layer, reciprocal synaptic connections between an RS and basket cell created the inhibition-based gamma rhythm; the decay time of the basket cell inhibitory synapse determined the period of this oscillation. In the deep layer, the M-current in the IB cell axons paced the beta2 rhythm. We proposed in the model that these dynamic motifs combined to create the slower beta1 oscillation. During beta1 activity, the LTS interneurons (initially disruptive to the coexistent gamma and beta2 oscillations) served a vital role, and the rhythm propagated between the cortical layers. Thus, unlike the coexistent gamma and beta2 rhythms, the beta1 rhythm represented a state in which activity in both deep and superficial layers of neocortex temporally combined to produce oscillations in which interlaminar interactions were vital.

We considered four manipulations of the model and in vitro preparation during beta1 activity. The outcome of each manipulation—separating the cortical layers, blocking the h-current, blocking IPSPs, or blocking the NMDA synapses between IB cells—was the same: elimination of the beta1 rhythm. We noted that the loss of beta1 activity occurred when the rhythm could not propagate between the cortical layers; thus, connections between layers were essential to the beta1 rhythm. We also noted that the beta1 rhythm is not quite a concatenation of the gamma and beta2 rhythms. Instead, we found that two different mechanisms support the beta2 and beta1 oscillations. In the high kainate condition, the beta2 rhythm resulted from antidromic activity in the IB cell axons. In the subsequent low kainate drive condition, the beta1 rhythm resulted from orthodromic activity in the IB cell dendrites.

We noted that the initial interval of coexistent gamma and beta2 activity must precede the beta1 rhythm. If we start the in vitro slice preparation in the low kainate drive condition (i.e., with kainate+NBQX) we find no oscillatory activity. Therefore, the initial interval of fast rhythms must change the network to support the slower beta1 oscillation. In the model, we assumed that the coexistent fast rhythms facilitated a potentiation of NMDA synapses between the population of IB cells [28]. In developing the computational model, we also proposed two additional scenarios for the change in network connectivity that supports the beta1 rhythm. First, we suggested strengthening the descending synapses from the superficial RS cells to the deep layer IB cells. Second, for the reduced model, we strengthened ascending synapses from the deep layer IB cells to the superficial inhibitory neurons. Each model was sufficient to generate the beta1 activity through period concatenation and agreed with the observational data. In vitro the transition to beta1 may perhaps incorporate aspects from all three scenarios, and future studies may suggest more general conditions for period concatenation incorporating all three models.

In the population models proposed in this work, we considered the activity generated in a single cortical column of somatosensory cortex. An improved model would describe the activity of multiple, interacting cortical columns. Including interactions between columns would permit synaptic connections not present in the single-column model (for example, excitatory synapses from deep layer IB cells to superficial RS cells observed in rat motor cortex [30]). Multi-column models incorporating adjacent cortices (and their region-specific oscillatory circuits and agonists [31]) may reveal even richer phenomena.

What computational roles might the separate gamma and beta2 rhythms, and the transition to a slower beta1 oscillation, serve? In the in vitro observations and computational models considered here, the initial faster rhythms coexisted in different cortical layers. The transition to the slower beta1 oscillation established a common rhythm that propagated between both layers. We might therefore interpret the transition to the beta1 oscillation as binding the superficial and deep layers of a cortical column. This binding organizes subnetworks of neurons within the input (superficial) and output (deep) layers of a cortical column [32]. Transitions between gamma, beta2, and beta1 oscillations are also observed in vivo [33],[34] although the role of rhythms in these cognitive functions remains unknown. However, beta oscillations (12–29 Hz) have been associated with long-range synchronization [35], which may be relevant to the interlaminar beta1 oscillation analyzed here.

Recent observations suggest that rhythms within distinct frequency intervals interact, and that these interactions may occur in different ways [36]–[45]. Given the increasing amount of evidence supporting cross-frequency interactions, the mechanisms involved in generating multi-frequency oscillatory states at the cellular and network level become fundamental to understanding brain rhythms. In this work, we described these mechanisms for a particular example of rhythm generation through period concatenation. Because the slow rhythm (beta1) resulted from the concatenation of two faster rhythms (beta2 and gamma), the fast activity was necessarily locked to a specific phase of the beta1 rhythm. For example, the interval between the basket cell firing and the RS (or LTS) cell firing defines (approximately) one gamma cycle. This fast activity is locked to a particular phase of the IB cell population bursts; this locking is apparent in Figure 8B. Analysis of these data alone suggests that the slow rhythm (beta1) modulates the faster rhythm (gamma). One might therefore conclude that the slow oscillation drives the faster rhythms. This conclusion is only partially correct; in the model the faster rhythms combine to create the slower oscillation. Data analysis alone cannot distinguish the mechanisms producing each rhythm. Instead, a combined approach of data analysis and biophysical modeling is required. Using this combined approach we suggest that faster rhythms, of different coexistent frequencies in different cortical laminae, may be present to maintain independent temporal processing of cortical input in each lamina. If synaptic potentiation occurs then these faster rhythms may concatenate, producing a slower rhythm representing the temporally correlated activity patterns in both deep and superficial laminae—thus uniting previously independent, lamina-specific activity patterns into a single cortical dynamic process.

## Methods

### Experimental Methods

Slices of parietal neocortex (450 µm thick), were prepared from adult male Wistar rats and maintained in an interface chamber. Details of artificial cerebrospinal fluid composition and recording techniques are in [18]. Spectra were all calculated from 60 s epochs of LFP data using MATLAB. All drugs were applied directly to the slice perfusate. All procedures were performed in accordance with the UK animals (scientific Procedures) act.

### Modeling Methods

The model consists of four cell types in two cortical layers. We describe the cells, synapses, and analysis methods in this section (detailed equations may be found in the Text S1. The model cells are reductions [46] of detailed multi-compartment representations of rat cortical neurons [15]. To construct the reduced model, we first include explicitly the mechanisms essential for gamma and beta2 rhythm generation observed in the superficial and deep cortical layers, respectively. We then include an additional cell type observed in vitro and chemical synapses to connect the two cortical layers. We begin with a description of the superficial layer model.

### Superficial Layer Model

A simple model of the superficial layer gamma rhythm consists of two neurons—one excitatory and one inhibitory—interacting through reciprocal synapses (the so-called Pyramidal-Interneuron-Network-Gamma or PING rhythm [12].) For the excitatory neuron, we implement a reduction of a superficial regular spiking (RS) pyramidal cell [15]. The reduced model consists of one compartment with four intrinsic membrane currents: a leak current, a transient inactivating sodium current (NaF current), a delayed rectifier potassium current (KDR current), and a hyperpolarization activated (or anomalous rectifier) current (h-current). The first three currents facilitate the generation of action potentials (or spiking) in the model [47]. The last current slowly depolarizes the RS cell following inhibitory input [48] and serves a vital role in the beta1 oscillation, as we describe in the Results section. The form of these intrinsic membrane currents, the reversal potentials, and the dynamics of the gating variables follow [49]. For the inhibitory neuron, we implement a superficial basket cell [15]. The reduced model consists of one compartment with three intrinsic membrane currents: a NaF current, a KDR current, and a leak current. The dynamics and reversal potentials for these currents follow the intrinsic interneuron properties stated in [50]. We note that the traditional spiking currents (the NaF, KDR, and leak currents) are similar (but not identical) for the RS cell and basket cell models.

We connect the RS and basket cells with reciprocal synapses. The equations governing the synaptic dynamics are similar to those described in [35] and [51]. The rise and decay times of the fast excitatory (AMPA) synapse are 0.25 ms and 1 ms, respectively. The rise and decay times of the fast inhibitory (GABA_A_) synapse are 0.5 ms and 5 ms, respectively. We also include a fast inhibitory autapse on the basket cell with the same GABA_A_ dynamics.

We did not include fast rhythmic bursting (FRB) neurons in the reduced gamma model. Recent experimental and modeling results suggest that these cells provide excitation via axonal plexus activity to drive neocortical gamma rhythms [50]. To avoid the complexity of such a system (which requires a large cell population with axonally interconnected principal cells) while maintaining sufficient phasic drive to generate gamma activity, we include tonic input currents injected to both cells. Each cell also receives a weak stochastic input with normal distribution that results in fluctuation of ±0.25 mV.

The final cell type we include in the superficial layer is a low threshold spiking (LTS) interneuron [21]–[23]. Our reduced model of this cell consists of a single compartment with four currents: a NaF current, a KDR current, a leak current, and an h-current. The dynamics and reversal potentials for these currents follow the intrinsic interneuron properties stated in [50]. We form reciprocal synaptic connections between the LTS interneuron and RS cell. The rise and decay times of the excitatory synapse are 2.5 ms and 1.0 ms, respectively, and the rise and decay times of the inhibitory synapse are 0.5 ms and 20 ms, respectively. We also include an inhibitory synapse from the basket cell to the LTS interneuron (rise time 0.5 ms and decay time 6.0 ms) and an inhibitory autapse on the LTS interneuron (rise time 0.5 ms and decay time 20 ms). A weak stochastic input (normally distributed) perturbs the voltage and results in fluctuation of ±0.25 mV.

To establish a population model of the superficial layer gamma activity, we create twenty replications of the RS-basket-LTS cell circuit described above and shown in Figure 2B. We make all parameters identical for each cell type of the population except for the depolarizing input currents which we independently vary for each cell of each triad. This heterogeneity causes the twenty RS cells to spike at different frequencies, ranging from 30 Hz and 50 Hz. We connect the RS-basket-LTS cell triads with a single type of connection: all-to-all electrical coupling between the RS cells [15].

### Deep Layer Model

To model the deep layer beta2 rhythm, we implement a reduction of the LV tufted intrinsically bursting (IB) pyramidal cell [15]. This cell type appears to dominate the beta2 rhythm in vitro [18]. Our reduced model of the single IB cell consists of four compartments: an axon, soma, apical dendrite, and basal dendrite. Each compartment contains the intrinsic membrane spiking currents: a NaF current, a KDR current, and a leak current. In addition, we include in the axon a muscarinic receptor suppressed potassium current (M-current [52],[53]), and in the dendrites we include a h-current, a M-current, and a high-threshold noninactivating calcium current (CaH current). The maximum conductance of the h-current in the apical dendrites exceeds that in the basal dendrites (to crudely mimic the increase in h-current observed with distance from the soma in apical dendrites [24],[25]). Both dendritic compartments also receive inhibitory synaptic input—the basal dendrite from a Poisson source of IPSPs and the apical dendrite from the superficial layer, as we describe below.

We connect the dendritic and axonal compartments to the soma through electrotonic coupling. The coupling conductances between the axon and soma were identical for both compartments. We set the coupling conductance from the dendrites to soma to exceed the coupling conductance from the soma to dendrites (so that the soma voltage has a weaker effect on the dendritic compartment's dynamics [46],[54].) Each compartment receives a tonic drive, and the axon and dendrites receive weak stochastic inputs (normal distribution, ±0.1 mV fluctuations).

To establish a population model of the deep layer activity we create twenty replications of the IB cell. Each member of the IB cell population consists of the same (four) compartments and currents described above. We make all parameters the same in each cell, except for the depolarizing currents to each compartment. We vary these inputs from cell to cell to establish heterogeneous bursting activity in the cell population; the interburst frequency of the twenty IB cells ranges from 20 Hz to 30 Hz. In the model, consisting of only twenty IB cells, we cannot represent a sparsely connected axon plexus which involves a large number of cells [15]. Therefore, we simply connect the IB cell population with all-to-all axonal gap junctions.

### Interlaminar Connections

From the superficial to deep layer we include a synapse from a single LTS interneuron to a unique apical dendrite of an IB cell (we note that the axons of superficial LTS interneurons extend up to layer I—a lamina rich in LV pyramidal cell apical dendrites [22].) Explicitly, if we number the population of LTS interneurons and IB cell apical dendrites 1 through 20, then LTS interneuron 1 forms a synapse on IB cell 1, LTS interneuron 2 forms a synapse on IB cell 2, and so on. The rise time and decay time of these synapses are 0.5 ms and 20 ms, respectively [15]. From the deep to superficial layer, we include synapses from each IB cell axon to all basket cells and LTS interneurons [55],[56]. For the basket cell postsynaptic target, the rise and decay times are 0.25 ms and 1.0 ms, respectively, and for the LTS interneuron postsynaptic target the rise and decay times are 2.5 ms and 50 ms, respectively. We note that the differences in the synaptic rise times may result from differences in axonal conduction times or dendritic delays (resulting perhaps from differences in dendritic morphologies or differences in locations of synaptic contacts [27],[57].) We do not include ascending synapses from deep layer IB cells to superficial pyramidal cells because anatomical studies indicate few synapses between these populations within a cortical column [15],[56],[58]. In addition to these three types of permanent synapses, we include a fourth potentiating synapse: a slow (NMDA) excitatory synapse extending from each IB cell axon to all IB cell basal dendrites [26],[27]. The rise and decay times for this synapse are 0.5 ms and 100 ms, respectively. We employ the latter synapse to generate the beta1 rhythm, as we show in the Results section.

### Numerical Methods

We use the Interactive Data Language (IDL) to compute numerical solutions to the model equations. We implement a second-order difference method with a time step of 0.01 ms, and follow the Euler-Maruyama algorithm to include stochastic inputs. Readers may obtain the simulation code by contacting the authors.

### Analysis Methods

To analyze the simulation results we compute two measures: the power spectrum and the cross-correlation. We briefly describe each measure here (detailed discussions of these measure may be found in the literature, for example [59]). To compute a power spectrum, we first compute ten realizations of the model, each with a different set of random tonic input currents to the cells and compartments, and each lasting 500 ms. Then for each realization we compute the population average voltage of the cell type of interest by summing the voltages of all (twenty) cells and dividing by the total number of cells. We then compute the power spectra of the population average voltages, and average these spectra across the ten realizations.

To compute the cross-correlation between the spike times of the RS cells and IB cell axons, we first simulate the model to create 1 s of data. We then locate the spike times and create new “binary” time series for each (of the twenty) RS cell and IB cell axon. We make the binary times series 0 everywhere except at the spike times where we set the time series to 1. We then compute the cross-correlation between the binary time series of each IB cell axon and the corresponding RS cell. By corresponding, we mean the RS cell in the unique triad whose LTS interneuron forms a synapse on a particular IB cell dendrite. We compute the cross-correlation in this way to avoid the effects of correlations in the subthreshold membrane potentials (e.g., correlations in hyperpolarizations of two cells.) We compute these cross-correlations for all twenty pairs of IB and RS cells, and average the results over the twenty cell pairs.

## Supporting Information

Text S1 Mathematical equations.
(0.04 MB PDF)
