# Supplementary material for: Rhythm Generation through Period Concatenation in Rat Somatosensory Cortex
Source: PLoS Comput Biol. 2008 Sep 5;4(9):e1000169. doi: 10.1371/journal.pcbi.1000169 (PMC2518953; doi:10.1371/journal.pcbi.1000169)
Supplement: Text S1 — Mathematical equations. (0.04 MB PDF) [file pcbi.1000169.s001.pdf]

Text S1. Supporting Information

Rhythm Generation through Period Concatenation in Rat Somatosensory Cortex

M. A. Kramer, A. K. Roopun, L. M. Carracedo, R. D. Traub, M. A. Whittington, and N. J. Kopell

In this section we present the mathematical formulations of the four cell types and synapses. Each set of equations follows the same format, as we now describe. We divide the current balance equation into three lines. In the first line (a) we list the tonic input current to the cell and the traditional spiking currents (the leak current, transient sodium current, and delayed rectifier current). In the second line (b) we list the other intrinsic currents of the cell (e.g., the h-current or M-current). If no additional intrinsic currents occur, then we list this line, but leave it blank. In the third line (c) we list the synaptic input currents to the cell and the current inputs from neighboring compartments, where appropriate. Following the current balance equation, we list the differential equations for each gating variable of the cell. Please contact the authors to obtain the simulation code.

Superficial RS pyramidal cell

$$C\dot{V}_e = -J_e - (70+V_e) - g_{\mathrm{NaF}_e} m_0[V_e]^3 h_e(-50+V_e)$$

$$- g_{\mathrm{KDR}_e} m_e^4 (95+V_e) \quad (1a)$$

$$- g_{\mathrm{AR}_e} m_{\mathrm{AR}_e}(35+V_e) \quad (1b)$$

$$- g_{ie} s_{ie}(80+V_e) - g_{\mathrm{L}_e} s_{\mathrm{L}}(80+V_e) - g_{\mathrm{gj}_e} GJ_e \quad (1c)$$

$$\dot{h}_e = \alpha_h[V_e](1-h_e) - \beta_h[V_e]h_e \quad (1d)$$

$$\dot{m}_e = \alpha_m[V_e](1-m_e) - \beta_m[V_e]m_e \quad (1e)$$

$$\dot{m}_{\mathrm{AR}} = \alpha_{\mathrm{AR}}[V_e,V_0](1-m_{\mathrm{AR}_e}) - \beta_{\mathrm{AR}}[V_e,V_0]m_{\mathrm{AR}_e} \quad (1f)$$

For this neuron (and those excitatory neurons that follow) we set the activation variable of the fast sodium current to the steady state value $m_0[V_e]$ defined in the *Auxiliary Functions* below. We include an h-currrent (1b) in this cell with dynamics similar to those stated in [1]; in this model, we multiply the forward rate function, α_{AR} in (1f), by a factor of 1.75 and multiply the backward rate function, β_{AR} in (1f), by a factor of 0.5. The effect of these changes is to accelerate the forward rate dynamics and slow the backward rate dynamics, respectively. We fix the inflection point of the sigmoid function $V_0 = 87.5$ mV (compared to the value $V_0 = 75.0$ used in [1]). The effect

of this change is to decrease the voltage at which the h-current activates. The superficial RS cell receives synapses (1c) from the superficial basket cell (with maximum conductance g_{ie}) and from the superficial LTS interneuron (with maximum conductance g_{L_e}). We include electrical coupling between each RS cell and all other RS cells. We denote this coupling in the model equations by the term GJ_e in (1c), and indicate the maximal conductance of this coupling by the term g_{gj_e}.

We set the parameters as follows: $J_e = -10.5(1.0)$, $g_{\mathrm{NaF}_e} = 200.0$, $g_{\mathrm{KDR}_e} = 20.0$, $g_{\mathrm{AR}_e} = 25.0$, $g_{ie} = 25.0$, $g_{\mathrm{L}_e} = 2.5$, and $g_{\mathrm{gj}_e} = 0.04$. Here, and in the definitions that follow, where two values are listed the first (second) term indicates the parameter value during high kainate (low kainate drive following potentiation) conditions.

Superficial basket cell

$$C\dot{V}_i = -J_i - (65 + V_i) - g_{\mathrm{NaF}_i} m_{0_i}[V_i]^3 h_i(-50 + V_i)$$

$$- g_{\mathrm{KDR}_i} m_i^4 (100 + V_i) \tag{2a}$$

$$\tag{2b}$$

$$- g_{ei} s_e V_i - g_{ii} s_{ii}(75 + V_i) - g_{ai} S_{ai} V_i \tag{2c}$$

$$\dot{h}_i = \alpha_{h_i}[V_i](1 - h_i) - \beta_{h_i}[V_i] h_i \tag{2d}$$

$$\dot{m}_i = \alpha_{m_i}[V_i](1 - m_i) - \beta_{m_i}[V_i] m_i \tag{2e}$$

For this neuron (and the inhibitory LTS interneuron) we set the activation variable of the fast sodium current to the steady state value $m_{0_i}[V_i]$. The superficial basket cell possesses the typical spiking currents (2a) and receives synaptic input (2c) from the superficial RS cell (with maximum conductance g_{ei}), from itself (with maximal conductance g_{ii}), and from the deep IB cell (with maximal conductance g_{ai}). The term S_{ai} represents the summed synapses from a population of twenty IB cells.

We set the parameters as follows: $J_i = 16.0(35.0)$, $g_{\mathrm{NaF}_i} = 200.0$, $g_{\mathrm{KDR}_i} = 20.0$, $g_{ei} = 1.0$, $g_{ii} = 20.0$, $g_{ai} = \{0.035, 0.055\}$ chosen uniformly for each deep IB cell to superficial basket cell connection.

Superficial LTS interneuron

$$
\begin{aligned}
C\dot{V}_{\mathrm{L}} =& -J_{\mathrm{L}} - g_{L_{\mathrm{L}}}(65+V_{\mathrm{L}}) - g_{\mathrm{NaF_L}} m_{0_i}[V_{\mathrm{L}}]^3 h_{\mathrm{L}}(-50+V_{\mathrm{L}}) \\
& - g_{\mathrm{KDR_L}} m_{\mathrm{L}}^4 (100+V_{\mathrm{L}}) && \text{(3a)} \\
& - g_{\mathrm{AR_L}} m_{\mathrm{AR_L}} (35+V_{\mathrm{L}}) && \text{(3b)} \\
& - g_{e\mathrm{L}} s_{e\mathrm{L}} V_{\mathrm{L}} - g_{i\mathrm{L}} s_{i\mathrm{L}} (80+V_{\mathrm{L}}) - g_{\mathrm{LL}} s_{\mathrm{L}} (80+V_{\mathrm{L}}) - g_{a\mathrm{L}} S_{a\mathrm{L}} V_{\mathrm{L}} && \text{(3c)} \\
\dot{h}_{\mathrm{L}} =& \alpha_{h_i}[V_{\mathrm{L}}](1-h_{\mathrm{L}}) - \beta_{h_i}[V_{\mathrm{L}}] h_{\mathrm{L}} && \text{(3d)} \\
\dot{m}_{\mathrm{L}} =& \alpha_{m_i}[V_{\mathrm{L}}](1-m_{\mathrm{L}}) - \beta_{m_i}[V_{\mathrm{L}}] m_{\mathrm{L}} && \text{(3e)} \\
\dot{m}_{\mathrm{AR_L}} =& \alpha_{m_{\mathrm{AR}}}[V_{\mathrm{L}},75](1-m_{\mathrm{AR_L}}) - \beta_{m_{\mathrm{AR}}}[V_{\mathrm{L}},75] m_{\mathrm{AR}_{LTS}} && \text{(3f)}
\end{aligned}
$$

This neuron possesses the typical spiking currents (3a) and an h-current (3b). For the dynamics of the h-current gating variables (3f), we use the forward and backward rate functions of [1]. The LTS interneuron receives four synaptic inputs: from the superficial RS cell, from the superficial basket cell, from itself, and from the deep IB cell (3c). We denote the maximal conductances as $g_{e\mathrm{L}}$, $g_{i\mathrm{L}}$, g_{LL}, and $g_{a\mathrm{L}}$, respectively. The term $S_{a\mathrm{L}}$ represents the summed synapses from a population of twenty IB cells.

We set the parameters as follows: $J_{\mathrm{L}} = 40.0(45.0)$, $g_{L_{\mathrm{L}}} = 6.0$, $g_{\mathrm{NaF_L}} = 200.0$, $g_{\mathrm{KDR_L}} = 10.0$, $g_{\mathrm{AR_L}} = 50.0$, $g_{e\mathrm{L}} = 2.0$, $g_{i\mathrm{L}} = 8.0$, $g_{\mathrm{LL}} = 5.0$, and $g_{a\mathrm{L}} = \{0.035, 0.045\}$ chosen uniformly for each deep IB cell to superficial LTS interneuron connection.

We now state the differential equations governing the four compartments (the apical dendrite, basal dendrite, soma, and axon) of the deep layer IB cell.

Deep layer IB cell apical and basal dendrite

$$
\begin{aligned}
C\dot{V}_d =& - J_d - g_{l_d}(70+V_d) - g_{\mathrm{NaF}_d} m_0[V_d]^3 h_d(-50+V_d) \\
& - g_{\mathrm{KDR}_d} m_d^4 (95+V_d) && \text{(4a)} \\
& - g_{\mathrm{CaH}_d} m_{\mathrm{CaH}_d}^2 (-125+V_d) - g_{\mathrm{KM}_d} m_{\mathrm{KM}_d}(95+V_d) \\
& - g_{\mathrm{AR}} m_{\mathrm{AR}_d}(25+V_d) && \text{(4b)} \\
& + g_{sd}(-V_d+V_s) - g_{ad} S_{ad} V_d - g_{\mathrm{L}d} s_{\mathrm{L}}(80+V_d) \\
& - g_{\mathrm{RAN}} s_{\mathrm{RAN}}(80+V_d) && \text{(4c)} \\
\dot{h}_d =& \alpha_h[V_d](1-h_d) - \beta_h[V_d] h_d && \text{(4d)} \\
\dot{m}_d =& \alpha_m[V_d](1-m_d) - \beta_m[V_d] m_d && \text{(4e)} \\
\dot{m}_{\mathrm{AR}_d} =& \alpha_{\mathrm{AR}_d}[V_d, V_0](1-m_{\mathrm{AR}_d}) - \beta_{\mathrm{AR}_d}[V_d, V_0] m_{\mathrm{AR}_d} && \text{(4f)} \\
\dot{m}_{\mathrm{KM}_d} =& \alpha_{\mathrm{KM}_d}[V_d](1-m_{\mathrm{KM}_d}) - \beta_{\mathrm{KM}_d}[V_d] m_{\mathrm{KM}_d} && \text{(4g)} \\
\dot{m}_{\mathrm{CaH}_d} =& \frac{1.0}{\tau_{\mathrm{CaH}}}(\alpha_{\mathrm{CaH}}[V_d](1-m_{\mathrm{CaH}_d}) - \beta_{\mathrm{CaH}}[V_d] m_{\mathrm{CaH}_d}) && \text{(4h)}
\end{aligned}
$$

The dendritic compartments of the IB cell consists of the typical spiking currents (4a), an h-current, M-current, and high-threshold calcium (CaH) current (4b). The dynamics of the h-current (4f) follow [1], and we multiply the forward and backward rate functions by 2.75 and 3.0, respectively. We set $V_0 = 75.0$ as used in [1], and note that we have increased the reversal potential to 25 mV in (4b). The dynamics of the M-current (4g) and CaH current (4h) follow directly from [1], and we multiply both the forward and backward rate functions of the latter by 3.0. The basal dendritic compartment receives synaptic input from the deep layer IB cells (maximal conductance g_{ad}) and from a Poisson source of IPSPs (maximal conductance g_{RAN}). The term S_{ad} represents the summed synaptic input from a population of twenty IB cells. The apical dendritic compartment receives synaptic input from a superficial layer LTS cell (maximal conductance $g_{\mathrm{L}d}$). Both dendritic compartments are coupled to the neighboring somatic compartment with conductance g_{sd}.

We set the parameters for both compartments as follows: $g_{l_d} = 2.0$, $g_{\mathrm{NaF}_d} = 125.0$, $g_{\mathrm{KDR}_d} = 10.0$, $g_{\mathrm{CaH}_d} = 6.5$, $g_{\mathrm{KM}_d} = 0.75$, $g_{sd} = 0.2$.

Five parameters differ for the apical and basal compartments. These are: J_d[apical]= 23.5(25.5), J_d[basal]= 23.5(42.5); g_{ad}[apical]= 0.0, g_{ad}[basal]= 0.0(0.04); g_{AR}[apical]= 155.0, g_{AR}[basal]= 115.0; g_{RAN}[apical]= 0.0, g_{RAN}[basal]= 125.0; and $g_{\mathrm{L}d}$[apical]= 4.0, $g_{\mathrm{L}d}$[basal]= 0.0.

Deep layer IB cell soma

$$CV̇_s = -J_s - (70 + V_s) - g_{\mathrm{NaF}_s} m_0[V_s]^3 h_s(-50 + V_s)$$
$$- g_{\mathrm{KDR}_s} m_s^4 (95 + V_s) \tag{5a}$$
$$\tag{5b}$$
$$+ g_{as}(V_a - V_s) + g_{ds}(V_d^a - V_s) + g_{ds}(V_d^b - V_s) \tag{5c}$$
$$\dot{h}_s = \alpha_h[V_s](1 - h_s) - \beta_h[V_s]h_s \tag{5d}$$
$$\dot{m}_s = \alpha_m[V_s](1 - m_s) - \beta_m[V_s]m_s \tag{5e}$$

The somatic compartment of the IB cell consists of typical spiking currents (5a) and is coupled to the neighboring dendritic (apical V_d^a, and basal V_d^b) and axonal compartments with maximal conductances of g_{as} and g_{ds}, respectively (5c).

We set the parameters as follows: $J_s = -4.5$, $g_{\mathrm{NaF}_s} = 50.0$, $g_{\mathrm{KDR}_s} = 10.0$, $g_{as} = 0.3$, and $g_{ds} = 0.4$.

Deep layer IB cell axon

$$C\dot{V}_a = -J_a - g_{L_a}(70 + V_a) - g_{\mathrm{NaF}_a} m_0[V_a]^3 h_a(-50 + V_a)$$
$$- g_{\mathrm{KDR}_a} m_a^4 (95 + V_a) \tag{6a}$$
$$- g_{\mathrm{KM}_a} m_{\mathrm{KM}_a} (95 + V_a) \tag{6b}$$
$$+ g_{sa}(V_s - V_a) - g_{\mathrm{gj}_a} GJ_a \tag{6c}$$
$$\dot{h}_a = \alpha_h[V_a](1 - h_a) - \beta_h[V_a]h_a \tag{6d}$$
$$\dot{m}_a = \alpha_m[V_a](1 - m_a) - \beta_m[V_a]m_a \tag{6e}$$
$$\dot{m}_{\mathrm{KM}_a} = \alpha_{\mathrm{KM}_a}[V_a](1 - m_{\mathrm{KM}_a}) - \beta_{\mathrm{KM}_a}[V_a]m_{\mathrm{KM}_a} \tag{6f}$$

The axonal compartment of the IB cell consists of typical spiking currents (6a) and an M-current (6b). The dynamics of the M-current gating variable (6f) follow [1] and we multiply the forward and backward rate functions by 1.5 and 1.25, respectively. The axon is coupled to the neighboring somatic compartment with maximal conductance g_{sa}. Each axon is also coupled — via gap junctions — to all other axons in the IB cell population. We denote this coupling in the model equations by the term GJ_a in (6c), and indicate the maximal conductance of this coupling by the term g_{gj_a}.

We set the parameters as follows: $J_a = -6.0(-0.4)$, $g_{L_a} = 0.25$, $g_{\mathrm{NaF}_a} = 100.0$, $g_{\mathrm{KDR}_a} = 5.0$, $g_{\mathrm{KM}_a} = 1.5$, $g_{sa} = 0.3$, $g_{\mathrm{gj}_a} = 0.002$.

Synapses

Each synapse evolves according to:

$$\dot{s_x} = -\frac{s_x}{\tau_{d_x}} + \frac{1-s_x}{\tau_{r_x}}(1+\tanh(\frac{V_{\mathrm{PRE}}}{10})) \tag{7}$$

where τ_{d_x} is the decay time for synapse x, τ_{r_x} is the rise time for synapse x, and V_{PRE} is the voltage of the presynaptic cell [2, 3]. We list in Table I the values for the decay and rise time of each synapse.

TABLE I: Synaptic rise and decay times. The rows and columns indicate the presynaptic and postsynaptic cell types, respectively. When a pair of numbers is listed, the first is the synaptic rise time and the second is the decay time. When no number is listed, no synapse connects row-column pair.

	**RS pyramidal cell**	**Basket cell**	**LTS interneuron**	**IB cell**
**RS pyramidal cell**	0.25 / 1.0	0.25 / 1.0	2.5 / 1.0	—
**Basket cell**	0.5 / 5.0	0.5 / 5.0	0.5 / 6.0	—
**LTS interneuron**	0.5 / 20.0	—	0.5 / 20.0	0.5 / 20.0
**IB cell**	—	0.25 / 1.0	2.5 / 50.0	0.5 / 100.0

Auxiliary Functions

Numerous auxiliary functions occur in the equations. These include m_0 and m_{0_i} and $\alpha_x[V]$ and $\beta_x[V]$. We define the latter two functions (the forward rate function and backward rate function, respectively, for current x) in terms of the equilibrium function ($m_\infty[V]$ or $h_\infty[V]$) and time constant ($\tau_m[V]$ or $\tau_h[V]$).

NaF and KDR currents for excitatory cells and compartments [1]:

$$m_0[V] = 1/(1+\exp((-V-34.5)/10)) \tag{8a}$$

$$h_\infty[V] = 1/(1+\exp((V+59.4)/10.7)) \tag{8b}$$

$$\tau_h[V] = 0.15+1.15/(1+\exp((V+33.5)/15)) \tag{8c}$$

$$m_\infty[V] = 1.0/(1.0+\exp((-V-29.5)/10)) \tag{8d}$$

$$\tau_m[V] = 0.25+4.35\exp(-||V+10||/10) \tag{8e}$$

NaF and KDR currents for inhibitory cells [4]:

$$m_{0_i}[V] = 1.0/(1.0+\exp((-V-38.0)/10.0)) \tag{9a}$$

$$h_{\infty_i}[V] = 1.0/(1.0+\exp((V+58.3)/6.7)) \tag{9b}$$

$$\tau_{h_i}[V] = 0.225+1.125/(1.0+\exp((V+37.0)/15.0)) \tag{9c}$$

$$m_{\infty_i}[V] = 1.0/(1.0+\exp((-V-27.0)/11.5)) \tag{9d}$$

$$\tau_{m_i}[V] = 0.25+4.35\exp(-||V+10.0||/10.0) \tag{9e}$$

AR current (or h-current) [1]:

$$m_{\infty_{\mathrm{AR}}}[V,V_0] = 1.0/(1.0+\exp((V+V_0)/5.5)) \tag{10a}$$

$$\begin{aligned}\tau_{m_{\mathrm{AR}}}[V] = 1.0/(&\exp(-14.6-0.086V)\\ &+\exp(-1.87+0.07V))\end{aligned} \tag{10b}$$

M-current: For this current we define the forward and backward rate functions [1].

$$\alpha_{\mathrm{KM}}[V] = 0.02/(1.0+\exp((-V-20)/5) \tag{11a}$$

$$\beta_{\mathrm{KM}}[V] = 0.01\exp((-V-43)/18) \tag{11b}$$

CaH current: For this current we define the forward and backward rate functions [1].

$$\alpha_{\mathrm{CaH}}[V] = 1.6/(1.0+\exp(-0.072(V-5)) \tag{12a}$$

$$\beta_{\mathrm{CaH}}[V] = 0.02(V+8.9)/(\exp((V+8.9)/5)-1) \tag{12b}$$

---

[1] Traub RD, Buhl EH, Gloveli T, Whittington MA (2003) Fast rhythmic bursting can be induced in layer 2/3 cortical neurons by enhancing persistent Na+ conductance or by blocking BK channels. J Neurophysiol 89:909–921.

[2] Kopell N, Ermentrout GB, Whittington MA, Traub RD (2000) Gamma rhythms and beta rhythms have different synchronization properties. Proc Natl Acad Sci USA 97:1867–1872.

[3] Gloveli T, Dugladze T, Rotstein HG, Traub RD, Monyer H, et al. (2005) Orthogonal arrangement of rhythm-generating microcircuits in the hippocampus. Proc Natl Acad Sci USA 102:13295–13300.

[4] Cunningham MO, Whittington MA, Bibbig A, Roopun A, LeBeau FEN, et al. (2004) A role for fast rhythmic bursting neurons in cortical gamma oscillations in vitro. Proc Natl Acad Sci USA 101:7152–7157.
